# Possible Electromagnetic Effects on Abnormal Animal Behavior Before an Earthquake

**DOI:** 10.3390/ani3010019

**Published:** 2013-01-10

**Authors:** Masashi Hayakawa

**Affiliations:** 1 Hayakawa Institute of Seismo Electromagnetics Co. Ltd., University of Electro-Communications (UEC) Incubation Center, 1-5-1 Chofugaoka, Chofu, Tokyo 182-8585, Japan; E-Mail: hayakawa@hi-seismo-em.jp; Tel.: +81-424-44-6349; Fax: +81-424-44-6349; 2 Advanced Wireless Communications Research Center, UEC, Chofu, Tokyo 182-8585, Japan; 3 Earthquake Analysis Laboratory, Kita-aoyama 2-12-42-R305, Minato-ku, Tokyo 107-0061, Japan

**Keywords:** abnormal animal behavior, earthquakes, seismogenic electromagnetic emissions

## Abstract

**Simple Summary:**

Possible electromagnetic effects on abnormal animal behavior before earthquakes.

**Abstract:**

The former statistical properties summarized by Rikitake (1998) on unusual animal behavior before an earthquake (EQ) have first been presented by using two parameters (epicentral distance (D) of an anomaly and its precursor (or lead) time (T)). Three plots are utilized to characterize the unusual animal behavior; (i) EQ magnitude (M) *versus* D, (ii) log T *versus* M, and (iii) occurrence histogram of log T. These plots are compared with the corresponding plots for different seismo-electromagnetic effects (radio emissions in different frequency ranges, seismo-atmospheric and -ionospheric perturbations) extensively obtained during the last 15–20 years. From the results of comparisons in terms of three plots, it is likely that lower frequency (ULF (ultra-low-frequency, f ≤ 1 Hz) and ELF (extremely-low-frequency, f ≤ a few hundreds Hz)) electromagnetic emissions exhibit a very similar temporal evolution with that of abnormal animal behavior. It is also suggested that a quantity of field intensity multiplied by the persistent time (or duration) of noise would play the primary role in abnormal animal behavior before an EQ.

## 1. Introduction

It is widely reported that land animals, birds, fish *etc*. often respond to earthquakes (EQs). A considerable number of books and papers have been published on this unusual biological behavior prior to EQs (e.g., [1,2,3,4]). Such abnormal animal behavior include: (i) disappearance of rats from a house; (ii) birds crying, *etc*. In addition to these publications, we can add recent works on this topic from the last 10 years or so [5,6,7,8,9,10,11,12]. The papers [7,8] have focused on the abnormal animal behavior for two disastrous EQs (Kobe and Wenchun EQs). These abnormal animal responses are generally called “macroscopic” anomalies of EQs, which are mainly based on anecdotal and retrospective records of animal behavior. Their studies enable us to deduce some physical insight into why and how animals react precursorily to seismic events. In his well-documented book, Rikitake (1998) [4] concluded that one of the most probable mechanisms of biological anomalies seems to be electromagnetic effects. On the other hand, electromagnetic phenomena associated with EQs (sometimes called seismo-electromagnetics) have been extensively investigated during the last 15–20 years for the sake of short-term EQ prediction, and the long-term observations have enabled us to yield some statistical results on different electromagnetic effects (e.g., [13,14]).

In this paper we first present some statistical conclusions by Rikitake (1998) [4] on macroscopic phenomena and then present recent statistical summaries of seismo-electromagnetic effects. Then we try to compare them in order to obtain some indication on the possible electromagnetic effects on biological systems before EQs. Finally we suggest that lower frequency (especially ULF (ultra-low-frequency, f ≤ 1 Hz) and ELF (extremely low frequency, f ≤ 1 kHz) seismogenic emissions, often recorded before an EQ, would be the most promising candidate to explain such unusual biological behavior.

## 2. Unusual Animal Behavior Before an EQ

In his book on “The science of macro-anomaly precursory to an earthquake”, Rikitake (1998) [4] summarized the behavior of animals (small and large), birds *etc*. on the basis of his extensive retrospective analyses for six large EQs including: (i) Ansei-Tokai EQ (M = 8.4, 1854); (ii) Nobi EQ (M = 8.0, 1891); (iii) Kanto EQ (M = 7.9, 1923); (iv) Toh-nankai EQ (M = 7.9, 1944); (v) Izu-oshima off-sea EQ (M = 7.0, 1978); and (vi) Miyagi-oki EQ (M = 7.4, 1978); and also publications by Kayano (1983, 1984) [15,16] on two EQs (Ibaraki-ken Nanbu EQ (M = 6.0, 1978) and Nagano-ken seibu EQ (M = 6.8, 1984)). His summary is presented in terms of two parameters: (1) distance of anomaly from the epicenter (D); and (2) precursory (or lead) time (T).

**Figure 1:**
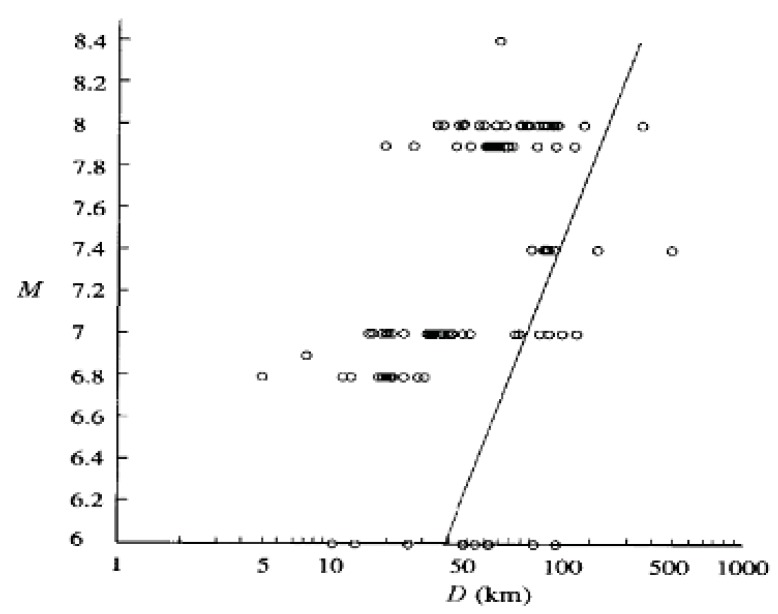
Dependence of unusual animal behavior on the earthquake (EQ) magnitude (M) and the epicentral distance (D). The straight log-log line is the averaged relation between M and D. Adapted from Rikitake (1998) [4] with permission of the publisher.

The statistical results of the macroscopic phenomena are presented in the following three common ways: (i) the relationship of the anomaly between the EQ magnitude (M) and the epicentral distance D; (ii) log T (in units of days) *versus* M; and (iii) the distribution of log T. Since the magnitude M is essentially a logarithmic scale, Figure 1 illustrates the log-log relation of abnormal animal behavior (among different macroscopic phenomena) between M and D [4]. Animals mean here dogs, cats and so on. This figure indicates a tendency that, for larger M values, the precursory anomalous of animal behavior is observed farther from the epicenter of a future EQ. The straight line in the figure—which traces the averaged relation between M-log D on the basis of various types of macroscopic effects (including animals, birds, fish, *etc*.) [4]—is expressed by

M = 1.86 + 2.6 log D(1)

**Figure 2:**
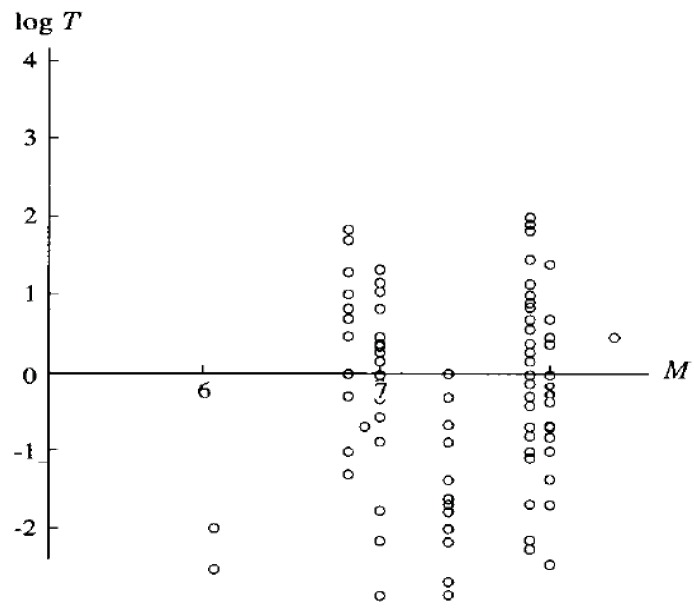
The relationship between M and log T (precursory time in units of days) for unusual animal behavior. Adapted from Rikitake (1998) [4] with permission of the publisher.

**Figure 3:**
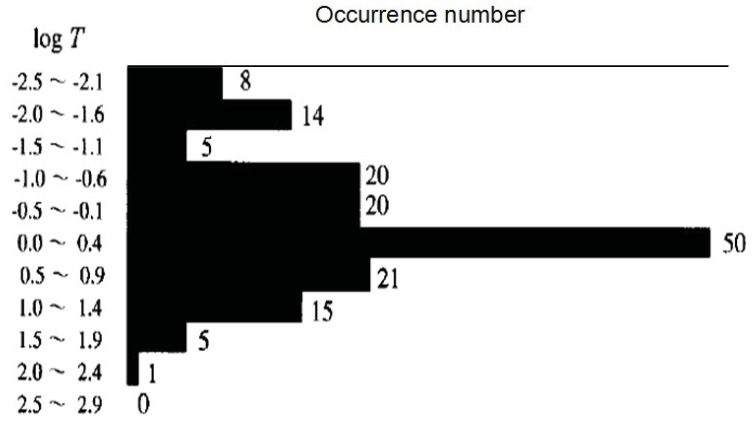
Occurrence histogram of time T (in units of days) of reported unusual animal behavior. Adapted from Rikitake (1998) [4] with permission of the publisher.

Figure 2 summarizes the precursor time (T) *versus* M relation. The value of T is distributed over a range from a few minutes to hundreds of days for any specific M. This suggests that there is no clear relationship between T and M. However, the occurrence histogram of log T (in units of days) in Figure 3 indicates that the distribution of T is concentrated in a range of T = 1–10 days.

Rikitake [4] added another informative statement. In response to the question whether there are any differences in the unusual behaviors between large and small animals, he reports that smaller animals seem to react earlier than larger animals. With respect to birds and fish, Rikitake [4] concluded that nearly all distribution of their unusual behavior is similar to those shown in Figure 1, Figure 2 and Figure 3. Similar results have been reported for snakes, earthworms, insects, *etc*.

As is seen from Figure 1 and Figure 2, Rikitake [4] did his analysis for relatively large EQs with M ≥ 7.0 because of the specific nature of the macroscopic pre-EQ anomalies. It is highly likely that the macroscopic data for lower M EQs would include more inaccurate information (or noise) to the macroscopic data for lower M values. At the same time, we need to emphasize here that most data on electromagnetic phenomena presented below were collected for M values smaller than 7 except for a few data on ULF emission, which tends to occur only for large EQs with M larger than 6–7.

## 3. Possible Sensory Mechanism of Animals

First of all, it seems highly plausible that animals behave unusually prior to EQs, Therefore, as the next step we have to ask what kind of stimuli are likely to lead to unusual animal behavior. Based on the extensive previous studies by Evernden (1976) [1], Buskirk *et al.* (1981) [2], Tributsch (1982) [3], and Rikitake [4], the following is a list of possible candidates of EQ precursory phenomena acting as stimuli:

(1) Change in atmospheric pressure
(2) Change in gravity
(3) Ground deformation (ground uplift and tilt change)
(4) Acoustic signals and vibrations due to the generation of microcracks
(5) Electromagnetic effects
(6) Ground water level change
(7) Emanation of gases and chemical substances

Based on the available evidence, Rikitake [4] concluded that the most probable candidate for abnormal animal behavior might be (5) electromagnetic effects, though some others, for example (4) and (7), cannot be ruled out. Recently Grant *et al.* [12] have discussed the effect of item (7).

## 4. Electromagnetic Effects and their Statistical Properties

The history of the study of seismogenic electromagnetic effects is rather short, on the order of a few decades, but there has been much progress with respect to short-term EQ prediction, especially since the 1995 Kobe EQ (e.g., see books [13,14,17,18,19,20] or review papers [21,22]). The observation of seismogenic effects can be customarily classified into two categories: (1) direct effect of electromagnetic emissions from within the lithosphere; and (2) indirect effects in the atmosphere or ionosphere. The summaries of different phenomena belonging to both categories will be discussed one by one in relation to the previous three common relationships of M *versus* D, T *versus* M, and occurrence histogram of T.

### 4.1. Seismogenic Radio Emissions

#### 4.1.1. DC Geoelectric Field

Based on long-term observations in Greece, Varotsos (2005) [23] has summarized his observation of SES (Seismic Electric Signal) activity; SES activity can frequently be detected in a short time interval of the order of one day. Large EQs then tend to take place about four weeks after such SES activity (*i.e.*, T = 4 weeks). With regard to the relationship of M *versus* D, Varotsos reported the following empirical relationship:

log (ED) = a M + b(2)

where E is the maximum amplitude of SES, and a and b are the constants determined empirically from the observational data. Equation (2) means that the SES intensity decreases with D as 1/D.

Compared to the lead time (T) of unusual animal behavior, the precursory time T of SES activity seems to be considerably larger as can be seen in Figure 3.

#### 4.1.2. ULF/ELF Electromagnetic Emissions

**Figure 4:**
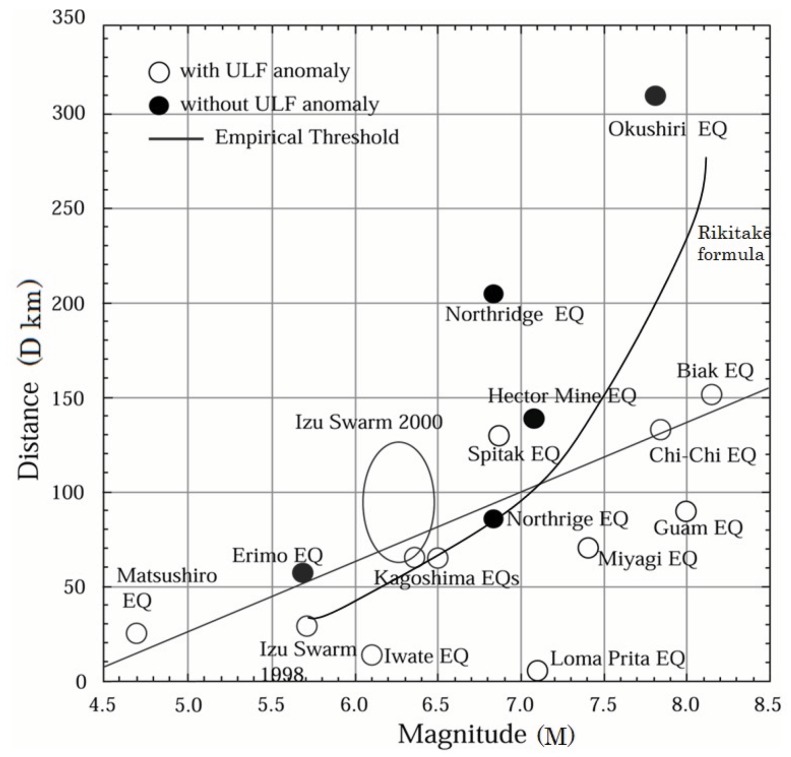
Summary of seismogenic ultra-low-frequency (ULF) radio emissions as M-D plot. Open circles indicate events with ULF anomaly, solid circles event without ULF anomaly. Straight line: empirical threshold (0.025 D = M − 4.5) by linear regression. Curve: Rikitake’s formula for unusual animal behavior.

This frequency range, especially ULF (f ≤ 1 Hz) has been extensively studied in different countries ever since three pioneering papers appeared for the Spitak [24], Loma Prieta [25] and Guam [26] EQs respectively. Hattori (2004) [27], Hayakawa and Hattori (2004) [28] and Molchanov and Hayakawa (2008) [14] summarized nearly all published data on seismogenic ULF emissions, plotted in Figure 4 in the form of D–M relations. The straight line, which marks the detection threshold of seismogenic ULF emissions (0.025 D = M – 4.5), is a linear regression line. According to these data, the maximum detection distance D is ~100 km for M = 7 and about 70–80 km for M = 6. The curve represents the empirical formula for abnormal animal behavior by Rikitake (1998) [4]. The straight line and the curve agree very closely for M values up to 7, but less so for the two events with M ≥ 7. This may simply be due to the fact that we used a linear regression line or to the fact that the number of events for M ≥ 7 is so small. When more events with M ≥ 7 become available, we will have to reexamine whether or not a present linear regression is acceptable.

Next we present the summary result of precursory time (T) of seismogenic ULF emissions based on the previous events as depicted in Figure 4 [29]. They show a typical temporal evolution.

(i) There seems to be no recognizable relationship between T and M.
(ii) ULF emissions show first an intensity enhancement 1–2 weeks before an EQ, lasting for about one week (at least a few days). Then there is evidence for quiescence a few days before an EQ. A pronounced increase occurs a few days before the EQ, followed by an additional abrupt increase a few hours before the EQ.

A typical example (f = 0.01 Hz) of such a temporal evolution of seismogenic ULF emissions can be found for the Loma Prieta EQ [25]. The intensity of the first peak is 20 nT/sqrt(Hz), and the imminent peak amounted up to 60 nT/sqrt(Hz).

Let us compare these results with the corresponding animal behavior in the previous section. The precursory time, T does not seem to be dependent on the EQ M, and the above summary on the temporal evolution seems to be very consistent with that of unusual animal behavior with the first peak at about 7–10 days before an EQ and the imminent peak just before the EQ. There is a conspicuous quiescence between the two peaks in the case of seismogenic ULF emissions, which looks to be in agreement with Figure 3 for the animal behavior. Of course, we are not sure whether a minimum in the distribution of T in Figure 3 indicates a real quiescence in the temporal evolution of animal behavior for a particular event or if it is merely a statistical combination of two separate peaks. Figure 4 in the form of D *versus* M for seismogenic ULF emissions is found to be very similar to the empirical formula by Rikitake (1998) [4] for animal behavior alone, especially in the M range below M = 7. Unfortunately ULF emission reports for large to very large EQs (M = 7–8) are extremely rare, so it is not possible to make a relevant comparison of the two.

Next we discuss the summary of ELF seismogenic emissions in the frequency range of less than 10 Hz. There have been very few reports on the radio emissions in this frequency range [29]. According to [14] and [29], based on a few years observation in Kamchatka, it is found that the radio emission in the frequency range from a few to a few tens of Hz appears to occur a few days before an EQ. Though no M–D relationship data are available, the detection distance D was found to be less than 300 km.

#### 4.1.3. ELF/VLF/LF radio noises

A significant number of papers have been published on radio emissions in these frequency ranges [30]. Once the radio emissions reach the atmosphere, they can propagate globally, with small attenuation, in the Earth-ionosphere waveguide (e.g., [31]). There have been very few reports on statistical studies on the characteristics of those seismogenic ELF/VLF radio emissions. Therefore, these frequency ranges are not quite suitable in the present paper with respect to a comparison with local unusual animal behavior.

**Figure 5:**
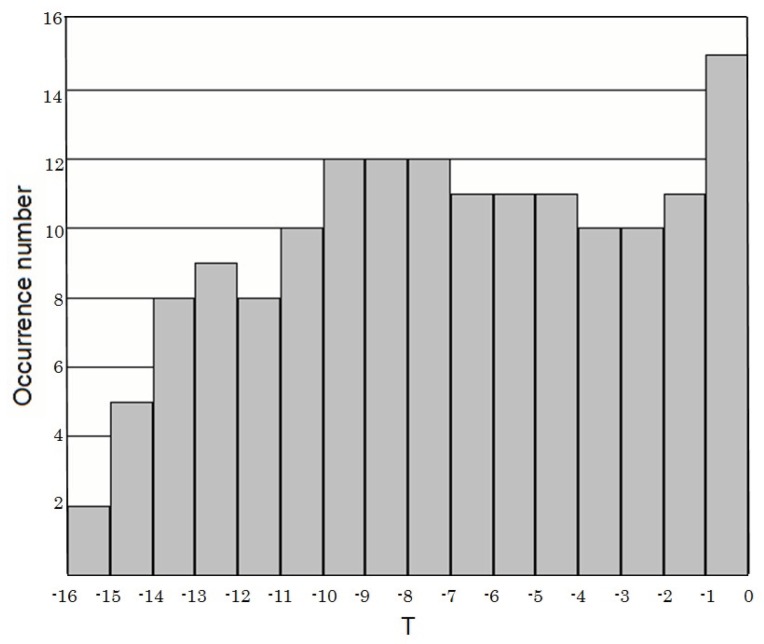
Occurrence histogram of pre-EQ extremely-low-frequency (ELF) radio emissions. Reproduced from a figure in Hata *et al.* (2006) [32] (with permission of the publisher) in which we define that strong emissions have a weight of unity and weak emissions have the weight of 0.5 (T in day).

**Figure 6:**
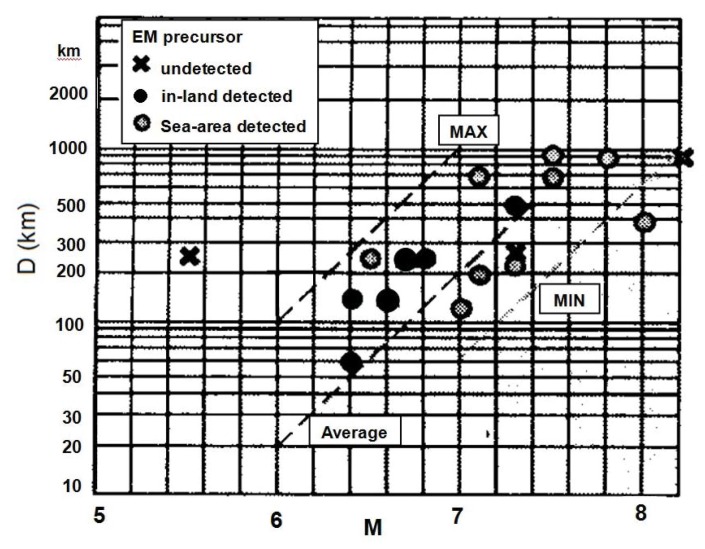
ELF radio noise plotted as M-D relation. Adapted from Hata *et al.* (2006) [32] with permission of the publisher.

However, there is one report on statistically relevant seismogenic ELF radio emissions (at 223 and 17 Hz) [32] drawing on observations over ten years. The precursory time (T) of these observations indicates that there is a peak in the occurrence 7–10 days before an EQ and an additional imminent peak just before the EQ (one day prior to the EQ), as shown in Figure 5. Figure 6 illustrates a statistical result on the M–D relationship, in which crosses indicate EQs with no ELF precursors, while the other symbols refer to EQs with ELF precursors. This figure shows that there is a general tendency that the detection distance (D) is larger for larger M values. The detection distance tends to be much larger for these frequencies than for the ULF emissions in Figure 4, due to the better propagation properties peculiar to this higher ELF range.

Additional studies on seismogenic VLF/LF radio emissions have been done [33,34]. However, the plots of M *versus* D and occurrence distribution of T are not available for comparison.

#### 4.1.4. HF/VHF Radio Emissions

In this frequency range, we can expect the radio emissions to be spatially localized. Enomoto *et al.* (1999) [35] presented a statistical study on seismo-HF/VHF radio emissions on the basis of observations at Tsukuba, Japan over the course of a few years. The following characteristics were obtained: (1) HF/VHF radio emissions appeared within 3–4 days before an EQ and an abrupt increase occurred within one day before the EQ; (2) the detection distance was found to be several tens of km from the observatory, so that the HF/VHF radio emissions seem to be indeed highly localized; (3) no M-D information is available.

### 4.2. Seismo-Atmospheric and Seismo–Ionospheric Effects

Two reviews have been published, one on seismo-atmospheric effect [30] and the other on seismo-ionospheric perturbations [36]. The most efficient tool to study seismo-atmospheric perturbations is the use of the over-the-horizon VHF transmitter signals. It was found that abnormal reception of over-the-horizon VHF signals takes place about one week before an EQ. However, the mechanisms are uncertain as to how such atmospheric anomalies may be generated, even though there are a few possible mechanisms including ground surface temperature anomalies or positive hole effects [37]. In this sense, it is rather difficult to connect this phenomenon with unusual animal behavior.

Nearly the same situation holds for seismo-ionospheric perturbations. Hayakawa (2009) [36] has concluded that the lower ionospheric perturbation might be observed about one week before an EQ. While, Liu (2009) [38] indicated that the upper ionospheric (F region) perturbations take place a few days before the EQ. Again, the type of mechanisms involved in the generation of seismo-ionospheric perturbation is poorly understood at the moment, though a few hypotheses have been proposed [36] such as: (1) radon emanation and the associated electric field change [39]; (2) positive hole effects and the corresponding electric field generation [37]; and (3) atmospheric oscillation effect [36]. In either case of (1) or (2), the generation of electric field (DC) is essentially of importance, so that it may be related with the abnormal animal behavior. The third possibility relates to the precursory ground movement, which seems to be ruled out as in Section 2 in the sense of abnormal animal behavior.

## 5. Discussions

Based on the suggestion of Rikitake (1998) [4], it appears that electromagnetic effects may be the most plausible candidate for causing abnormal animal behavior. In this paper, we first presented the statistical relations found by Rikitake between unusual behaviors of animals (dogs, cats, *etc*.) in terms of the three relationships of (i) M *versus* D, (ii) T *versus* M, and (iii) T occurrence histograms. Then we presented the statistical properties of seismogenic radio emissions in different frequency ranges while paying attention to the relationships (i)–(iii). Though the number of events for electromagnetic emissions is not large enough to have high statistical significance, it seems plausible as the result of comparisons, that the electromagnetic emissions in the ULF and lower ELF range are found to exhibit very similar characteristics in terms of the three relationships. Those emissions suggest a distinct temporal evolution: a first peak around one week before an EQ, followed by a second peak just before the EQ. This temporal change appears to be consistent with (or similar to) that of unusual animal behavior; the temporal evolution of unusual animal behavior also yields two peaks, a broad one about a week before an EQ and another just before the EQ.

In the field of experimental biology, a laboratory experiment has recently been attempted by Nishimura *et al.* (2010) [11], which appears to be worthwhile so as to understand our hypothesis presented in this paper. They have suggested that lizards are likely to perceive the low frequency electromagnetic signals. However, further expounded experiments are essential to have a better understanding of animal sensory perception because this kind of laboratory experiment is very time-consuming and obtaining any statistically reliable results is generally difficult.

It is further worthwhile to also mention studies of biological effects of radio signals by scientists in other disciplines. In the engineering EMC (Electromagnetic Compatibility) area, great attention has been paid to the possible biological effects of electromagnetic radiation in different frequency ranges (from ELF (power line frequencies) to VHF or even higher (mobile phone frequencies)) [40]. Since Werthemier and Leeper (1979) [41] noted a higher incidence of cancer among children living in homes where ELF exposure was presumed to be higher than usual, there has been a very large number of studies in different countries on the biological effect of ELF power lines [42]. Even after such extensive investigations, a consensus seems not to have been reached yet regarding the effect of power lines on biology effects (human, animals, *etc*.). However, when looking at the complete list of papers in the summary report by [40] on: (1) the exposure of ELF magnetic field on animals; and (2) the relationship between ELF magnetic field exposure and cancers, it appears that a considerable number of papers suggest some influence on smaller animals (like mice or rats), while the relationship between ELF exposure and human cancer remains quite uncertain. The serious problem in the EMC area is the statistical reliability or significance of the data because the number of samples is generally not sufficient. This is nearly the same situation as in the study of macroscopic anomalies of EQs.

Next we discuss the biological effect of natural radio emissions. Cases of changes in the natural electromagnetic fields can be found in the scientific literature linking to observable effects on higher life forms which can also be found in the scientific literature. Such natural processes include solar, geomagnetic, cosmic ray, lightning activity, *etc.*, and recently Cherry (2003) [43] suggested the importance of Schumann resonances in biology. Schumann resonances are a global resonance phenomenon excited primarily by background lightning discharges in the Earth-ionosphere waveguide [31]. The Schumann resonances are very weak, but very stationary with distinct frequencies at 8, 14, 20 Hz, *etc*. The high stationarity of Schumann resonances stands in sharp contrast to previously mentioned natural phenomena which are very transient. We have studied the biological effect of this Schumann resonance on the basis of our own ELF observation in Moshiri, Hokkaido and the simultaneous observation of human blood pressure, heart rate and depression, *etc*. [44]. These data were obtained between April and July 2001. It was found that the blood pressure in humans shows statistically significant mean differences between normal and enhanced Schumann resonance days. That is, the mean blood pressure rate is significantly lower for enhanced Schumann resonance days than for normal Schumann resonance days. The ELF magnetic field of Schumann resonances is extremely weak (<1 pT/sqrt(Hz)), so that its stationarity (or persistence) appears to be of primary importance.

Finally, we suggest that any ULF/ELF seismogenic radio emissions may be a dominant source of unusual animal behavior before an EQ. The intensity of seismogenic ULF/ELF emissions is on the order of 1–50 nT (1 nT corresponds to 0.3 V/m in the atmosphere). These values have been used in the EMC area, to derive, for the purpose of risk management, a maximum permissible exposure, i.e. the field intensity (either electric or magnetic) multiplied by the exposure time is thought to be the primary factor in studies of abnormal animal behavior. In other words, even though the field intensity may not be very large (such as seismogenic noises (Schumann resonance as well)), the persistence or prolongation of the radiation may play an essential role in animal behavior. For example, seismogenic precursory ULF emissions are known to persist, at least, for a few days or even up to about one week. Of course, there remain so many questions; e.g., is the electric or magnetic field influential on the animals? 

## 6. Conclusion

Based on the intense comparison of characteristics of abnormal animal behavior and seismogenic electromagnetic radiation in a wide frequency range, we come to the conclusion that lower frequency (such as ULF and ELF) electromagnetic emissions are a plausible candidate to explain abnormal animal behavior before an EQ. In order to verify this hypothesis, the following steps are essential: (i) further anecdotal and retrospective studies of abnormal animal behavior; (ii) a coordinated measurement of animal behavior with seismic and chemical sensors in combination with electromagnetic sensors in a seismically active region (as suggested in [5]).
